# COVID‐19‐induced granulomatosis with polyangiitis: A case report of a 16‐year‐old East Asian and literature review

**DOI:** 10.1002/iid3.70010

**Published:** 2024-09-06

**Authors:** Rong Jiang, Haibo Zhou, Long Wen, Xianglong Kong, Zhiguo Zhou

**Affiliations:** ^1^ Department of Respiratory and Critical Care Medicine Changsha Hospital of Xiangya School of Medicine Central South University Changsha Hunan China; ^2^ Department of Respiratory and Critical Care Medicine University of South China Hengyang Medical School Hengyang Hunan China

**Keywords:** case report, coronavirus disease 2019, granulomatosis with polyangiitis, literature review

## Abstract

**Objective:**

Antineutrophil cytoplasmic antibody (ANCA)‐associated vasculitis (AAV) is divided into granulomatosis with polyangiitis (GPA), microscopic polyangiitis, and eosinophilic GPA. It is one of the most severe and potentially fatal autoimmune inflammatory conditions. The etiology and pathology of AAV are complex and poorly understood. Since the onset of the Coronavirus Disease 2019 (COVID‐19) pandemic, numerous reports have documented GPA cases following COVID‐19, suggesting a potential link between COVID‐19 and the development of GPA. This case report discusses a 16‐year‐old East Asian boy who developed GPA with diffuse alveolar hemorrhage after contracting COVID‐19. Additionally, a literature review was conducted to gain a deeper understanding of this disorder.

**Methods:**

The study involved a retrospective analysis of the data of a case of GPA post‐COVID‐19 infection, aiming to summarize the clinical characteristics of GPA post‐COVID‐19 infection through a search of databases (PubMed, Wanfang Data, and CNKI), supplemented by standard searches in Google Scholar, Cochrane, Scopus, and LitCovid, and to conduct a comprehensive analysis of the literature.

**Results:**

A total of 12 cases were identified and, when combined with the present case, yielded 13 cases of GPA post‐COVID‐19 infection, comprising 5 males and 8 females with an average age of (40.6 ± 19.5) years. The interval between COVID‐19 infection and the diagnosis of GPA varied from 1 day to 3 months across all cases. Mortality was reported at 7.7% (1/13). The most common clinical manifestations included cough (69.2%) and dyspnea (46.1%). Computed tomography scans revealed ground‐glass opacities and multifocal pulmonary nodules. In all cases, positive findings for c‐ANCA and protease 3‐antibody were observed. Renal involvement was observed in more than half of the patients.

## 1 BACKGROUND

Granulomatosis with Polyangiitis (GPA) represents a potentially fatal multisystem disease characterized by necrotizing granulomatous vasculitis affecting the minor arteries and veins. This condition primarily involves the lungs, upper respiratory tract, and kidneys. Although the exact mechanisms underlying GPA development remain unclear, several factors, including infections, medications, and environmental exposures, are thought to contribute to the disease's pathophysiology through the induction of autoimmunity.^1^, ^2^ Since the onset of the Coronavirus Disease 2019 (COVID‐19) pandemic, numerous reports have documented antineutrophil cytoplasmic antibody (ANCA)‐associated vasculitis (AAV) cases following COVID‐19, suggesting a potential link between COVID‐19 and the development of GPA. We hereby present a case of new‐onset GPA with diffuse alveolar hemorrhage (DAH) in an adolescent patient following COVID‐19 infection. This represents the first documented case of GPA with DAH in an adolescent within Hunan province, China, subsequent to COVID‐19 infection. Additionally, we conducted a literature review on GPA post‐COVID‐19 infection, utilizing database searches to summarize the clinical characteristics of GPA in the context of COVID‐19.

## 2 CASE PRESENTATION

A 16‐year‐old male patient was admitted to the hospital with a 1‐month history of recurrent fever, cough, and phlegm. One month after contracting the novel coronavirus (SARS‐CoV‐2) (mild infection), the patient exhibited fever, cough, and sputum production, with a maximum temperature of 39°C. Additional symptoms included chills, paroxysmal cough, yellow pus sputum, fatigue, shortness of breath, palpitations, nasal bleeding, tinnitus, and nasal cartilage collapse. He did not experience headaches, dizziness, hemoptysis, night sweats, chest pain, chest tightness, nausea, vomiting, abdominal pain, diarrhea, or other discomforts. The patient was initially treated at the People's Hospital of Dong Autonomous County, Xinhuang, Hunan Province. A chest contrast‐enhanced computed tomography (CT) scan revealed infectious lesions with abscess formation in both lungs. Pathological results from a CT‐guided lung biopsy showed viable lung pathological tissues with areas of evident fibrous tissue hyperplasia and significant inflammatory cell infiltration. Despite a 10‐day course of ceftazidime injection (0.95 g, bid), the patient's condition did not improve. He was then referred to the First People's Hospital of Huaihua City, where a diagnosis of lung abscess was considered. The patient was treated with piperacillin‐tazobactam (4.5 g, Q8h) and vancomycin (0.5 g, Q8h) for 1 week, but continued to have recurrent fevers. The patient was referred to our hospital for further treatment.

## 3 PAST MEDICAL HISTORY

The patient had undergone bilateral knee surgery 4 years prior, though the specific etiology and surgical method were unknown. He denied any other significant medical history and had received three doses of the Sinovac vaccine. Notably, the patient had never been infected with SARS‐CoV‐2 before the current illness.

## 4 PHYSICAL EXAMINATION

Upon examination, the patient's vital signs were as follows: body temperature 37.6°C, respiratory rate 21 breaths per minute, blood pressure 97/60 mmHg, heart rate 102 beats per minute, and peripheral oxygen saturation 98%. The patient was conscious and exhibited a visible saddle nose deformity. Lung auscultation revealed a few moist rales, without any evidence of pleural friction. No abnormalities were identified in the heart or abdomen.

## 5 DIAGNOSTIC ASSESSMENT

### 5.1 Biochemical examination

Upon admission, a blood culture specimen was collected and yielded the following results: white blood cell (WBC) count 20.8 × 10^9^/L (normal range: 4–10 × 10^9^/L), with neutrophils accounting for 84% (normal range: 40%–75%); erythrocyte sedimentation rate (ESR) 112 mm/h (normal range: 0–20 mm/h); procalcitonin (PCT) 0.88 ng/mL (normal range: 0–0.01 ng/mL); C‐reactive protein (CRP) 247.8 mg/L (normal range: 0–6 mg/L). The liver and kidney function and coagulation routine of the patient were normal. His antineutrophil cytoplasmic antibody (c‐ANCA) and protease 3 (PR3) were positive. COVID‐19 nucleic acid test negative. Urinalysis revealed the presence of occult blood (10 cells/µL), protein (0.3 g/L), and red blood cells (0–2/HP).

### 5.2 Imaging examination

**Figure 1:**
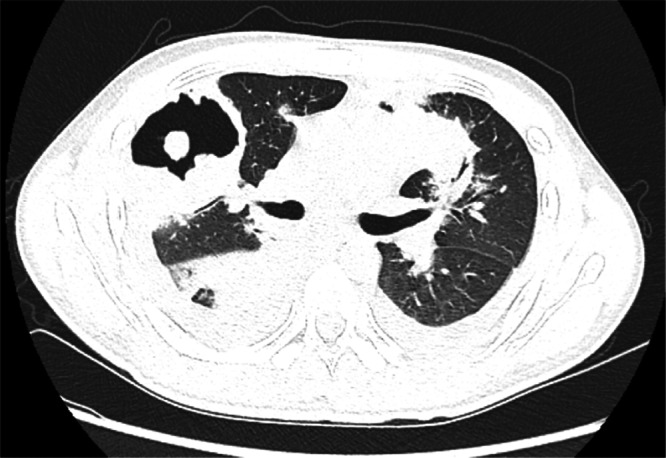
Chest computed tomography on admission. Chest computed tomography on admission showed multiple nodular shadows and cavities, creating “an island in the lung.”

A CT scan of the patient's paranasal sinuses revealed bilateral maxillary sinusitis and left frontal sinusitis. Subsequent CT scans of the head and abdomen showed no obvious abnormalities. Pulmonary CT imaging revealed multiple nodules and cavities in both lungs, described metaphorically as “islands in the lung” (Figure 1). Additionally, right empyema and bilateral pleural effusion with a small amount of pericardial effusion were considered. Bronchoscopy indicated necrotic nodules in the right main bronchus. A biopsy of the left main bronchus revealed a small amount of mucosal tissue covered by respiratory epithelium with chronic inflammation, but no atypical epithelium. Bone marrow cytology was performed to elucidate the underlying cause of the patient's fever and exclude the possibility of hematological disorders. The bone marrow puncture findings indicated marked hyperplasia of the bone marrow, evident granulocytic shift and infection, and pronounced megakaryocytic hyperplasia.

### 5.3 Microbiology examination

Metagenomic next‐generation sequencing (mNGS) was applied to analyze alveolar lavage fluid samples collected in the outer hospital. Initially, no bacteria, tuberculosis, or fungi were identified. However, after a 1‐month interval, both mNGS and culture of the alveolar lavage fluid revealed the presence of Acinetobacter baumannii.

### 5.4 Diagnosis and differential diagnosis

The patient had nasal bleeding, tinnitus symptoms, nasal cartilage involvement (visible in the nose), cytoplasmic antineutrophilic cytoplasmic antibodies (c‐ANCA) and anti‐PR3 antibodies positive, chest images showed pulmonary nodules and cavities, and sinus CT indicated sinusitis. According to the 2022 edition of the American College of Rheumatology ACR/European League against Rheumatism EULAR vasculitis classification criteri,^3^ the GPA diagnosis is consistent. The clinical and radiologic findings of DAH and ANCA‐positive rapidly progressive glomerulonephritis should also be distinguished from microscopic polyangiitis, Goodpasture syndrome, and systemic lupus erythematosus characterized by pulmonary and renal manifestations.

### 5.5 Treatment and outcomes

Upon admission to our medical facility, the patient was administered Latamoxef (1 g, Q8h for 3 days) and Teicoplanin (0.4 g, Q8h for 3 days), followed by Meropenem (1.0 g, Q8h for 7 days) and Linezolid (600 mg, Q12h for 7 days). Additionally, a 40 mg dose of methylprednisolone was given daily, and the patient received an infusion of immunoglobulin of 10 g per day for 3 days. Despite these treatments, the patient continued to experience fever and shortness of breath upon exertion. Approximately 2 weeks after treatment initiation, the patient developed respiratory distress and hemoptysis (500 mL). Vital signs included a heart rate of 132 beats per minute, a respiratory rate of 41 breaths per minute, and an oxygen saturation of 80%, a significant decline from prior readings. A diagnosis of GPA, acute respiratory distress syndrome, and DAH syndrome was considered, and mechanical ventilation with endotracheal intubation was initiated. Given the reproductive toxicity of cyclophosphamide, a treatment regimen of rituximab (0.5 g weekly) and plasma exchange was selected, along with methylprednisolone injections (0.5 g intravenously for 3 days). Alveolar lavage fluid analysis revealed the presence of Acinetobacter baumannii. The patient was treated with cefoperazone‐sulbactam (3 g, Q8h) and minocycline capsules (0.1 g, Q12h) for antiinfection, compound sulfamethoxazole tablets (2 tablets, bid) to prevent Pneumocystis jirovecii pneumonia, and itraconazole capsules (200 mg, once daily) to prevent fungal infection. The patient's symptoms of fever and hemoptysis showed significant improvement, and oxygenation levels increased. The trachea was successfully extubated 5 days after intubation. However, the patient developed a pneumothorax without an apparent cause and was treated with closed drainage in the right thoracic cavity. Two days later, the patient experienced an acute exacerbation of respiratory distress (respiratory rate of 38 breaths/min), a decline in oxygen saturation (SpO2 60%), and required reintubation. Laboratory investigations revealed a WBC count of 30.86 × 10⁹/L (normal range: 4–10 × 10⁹/L), an elevated PCT level (51 ng/mL), and high inflammatory markers, including CRP (118.6 mg/L). The patient exhibited new nasal damage and worsening hearing loss. The ESR was 121 mm/h (normal range: 0–15 mm/h), and CRP was 118.6 mg/L (normal range: 0–5 mg/L), indicating aggravation due to uncontrolled vasculitis rather than a single infection. Treatment included methylprednisolone injection (40 mg/day) and rituximab injection (0.5 g weekly), with an adjusted antibiotic regimen of cefoperazone‐sulbactam injection (1.0 g, Q8h) and linezolid injection (0.6 g, Q12h). The patient also received itraconazole capsules and a combination of sulfamethoxazole tablets for antiinfection therapy. Despite these measures, the patient's condition remained uncontrolled, with oxygenation (oxygenation index less than 100) and blood pressure (less than 90/60 mmHg) persistently compromised. Bedside chest radiography revealed a significant right pneumothorax, necessitating closed thoracic drainage, which did not significantly increase blood oxygen levels. Bedside echocardiography showed a large right ventricle, a small left heart, pulmonary hypertension, and mild tricuspid regurgitation.

The patient was considered to have acute pulmonary embolism with shock and had indications for thrombolytic therapy, but there was also a risk of fatal hemoptysis after thrombolytic therapy. The family members accepted the worst outcome and signed to refuse thrombolytic therapy. Due to financial constraints, the patient's family did not opt for extracorporeal membrane oxygenation treatment. Unfortunately, the patient succumbed to acute pulmonary embolism with shock.

### 5.6 Search strategy and literature review

As of Jan 2024, we conducted a systematic literature review employing MeSH search terms “COVID‐19” or “Coronavirus Disease 2019” in conjunction with “Granulomatosis with Polyangiitis,” “GPA,” or “ANCA‐Associated Vasculitis” across databases such as PubMed, Wanfang Data, and CNKI, supplemented by standard searches in Google Scholar, Cochrane, Scopus, and LitCovid. Three authors (RJ, ZJZ, and HBZ) independently assessed the titles and full texts of all relevant articles. The three researchers analyzed the search outcomes and reached a consensus on the selection of articles for inclusion. *Inclusion criteria*: (1) GPA newly diagnosed after COVID‐19 infection. (2) Complete case data. *Exclusion criteria*: (1) GPA developed after COVID‐19 vaccination. (2) Not diagnosed with GPA, only considered for patients of AAV. 2. Incomplete clinical data. Data regarding age, gender, clinical manifestations, laboratory and imaging results, diagnostic approaches, therapeutic interventions, and the prognosis of GPA following COVID‐19 infection were extracted from the selected articles by the authors.

### 5.7 Results from the literature review

By January 2024, a total of 12 English‐language cases of GPA post‐COVID‐19 infection, with no cases reported in Chinese literature were retrieved.^4^, ^5^, ^6^, ^7^, ^8^, ^9^, ^10^, ^11^, ^12^, ^13^, ^14^, ^15^ Including our case, the total number of reported cases of GPA following COVID‐19 infection amounted to 13. The disorder predominantly affects females, with ages ranging from 16 to 72 years (the median age is 38 years), and the interval between COVID‐19 infection and GPA diagnosis spanned from 1 day to 3 months. Among the 13 patients (including our case), nine had no prior comorbidities, whereas two presented with diabetes and rhinitis as preexisting conditions.

Immunological testing revealed that PR3 antibodies and C‐ANCA were universally positive (100%) among the patients. The most frequently observed clinical manifestations included cough (69.2%) and dyspnea (46.1%). Pulmonary imaging studies revealed multifocal cavitary pulmonary nodules in seven patients, ground‐glass opacities in three, DAH in two, and pleural effusions in three. Renal involvement was observed in 58.3% (7/12) of the patients. Of those, renal biopsies were performed in four, revealing glomerular necrosis and crescental lesions in three. Hemodialysis was performed in only two patients. The mortality rate was documented at 7.7% (1/13) (Table 1). All 13 patients received glucocorticoid therapy, the standard treatment for vasculitis. Ten patients underwent rituximab therapy, and two underwent plasmapheresis. At follow‐up, 12 patients demonstrated symptomatic and radiographic improvement (Table 2).

**Table 1:** Summary of clinical findings, demographics, and treatment strategies of GPA after COVID‐19.

Number	Case	Age (years), sex	Past medical history	Chronology with COVID‐19	GPA treatment	Outcomes	Kidney involvement and kidney biopsy	Haemodialysis
1	Nakamura et al.^4^	17, F	None	45 days	MPZ+RTX	Good prognosis	No	No
2	Weynand et al.^5^	51, F	diabetes, hypertension, osteoarthritis, and chronic nonallergic rhinitis	90 days	MPZ+RTX	Good prognosis	No	No
3	Selvarj et al.	60, F	dietcontrolled diabetes mellitus, allergic rhinitis	30 days	MPZ+RTX+PEX	Good prognosis	Yes, severe crescenteric and necrotizing glomerulonephritis	Yes
4	Lind et al.^7^	40, M	None	10 days	RTX+MPZ	Good prognosis	Yes, glomerular necrosis and crescental lesions	No
5	Izci et al.^8^	36, F	None	A few weeks	MPZ+CYC	Good prognosis	Yes, pauci‐immune necrotizing GN with cellular crescents	No
6	Bryant et al.^9^	16, F	asthma	7 days	MPZ+MMF	Good prognosis	No	No
7	Qurratulain et al.^10^	71, F	None	14 days	RTX+MPZ	Good prognosis	Yes (no renal biopsy	No
8	Bressler et al.^11^	46, M	None	14 days	MPZ+RTX	Good prognosis	Yes, interstitial mixed cell infiltrates and intraglomerular thrombi and necrosis	No
9	Giles et al.^12^	28, M	None	1 day	MPZ+RTX	Good prognosis	Not mentioned	No
10	Basnet et al.^13^	26, F	None	Not mentioned	RTX+MPZ	Good prognosis	Yes (no renal biopsy)	No
11	Romanello et al.^14^	41, M	None	90 days	RTX+MPZ	Good prognosis	No	No
12	Mandegar et al.	72, F	hypertension and ischemic heart disease	60 days	MPZ+methotrexate	Good prognosis	No	No
13	Our case	16, M	None	30 days	MPZ+RTX+PEX	Die	Yes (no renal biopsy)	Yes

Abbreviations: COVID‐19, Coronavirus Disease 2019; CYC, cyclophosphamide; GPA, granulomatosis with polyangiitis; MMF, mycophenolate mofetil; MPZ, methylprednisolone; PEX, plasmapheresis; RTX, rituximab.

**Table 2:** Clinical characteristics, laboratory and pulmonary CT imaging features of COVID‐19 induced GPA cases.

**Features**	**Total (*n*** = **13)**
Symptom, *n* (%)	
Fever	5 (38.4)
Cough	9 (69.2)
Dyspnea	6 (46.1)
Bleeding (dyspnea/alimentary tract hemorrhage)	3 (23.1)
Rash	4 (30.7)
Arthralgia/Myalgia	5 (38.5)
Laboratory features, *n* (%)	
C‐reactive protein↑	8 (61.5)
C‐ANCA(+)	13 (100)
PR3‐antibody(+)	13 (100)
Pulmonary CT imaging features, *n* (%)	
Ground glass opacities in thorax	3 (23.1)
Multifocal pulmonary nodules	7 (53.8)
Diffuse alveolar hemorrhage	2 (15.4)
Pleural effusions	3 (23.1)
Need for O_2_ support	3 (23.1)
Kidney involvement at presentation, *n* (%) (*n* = 12)	7 (58.3)
Mortality, *n* (%)	1 (7.7)

Abbreviation: CT, computed tomography.

## 6 DISCUSSION

Over the past 3 years, COVID‐19, caused by the SARS‐CoV‐2 virus, has emerged as one of the deadliest pandemics in recorded history. GPA is a systemic condition characterized by vasculitis in multiple organs, predominantly affecting individuals of Caucasian descent aged 40–65, with an incidence of approximately one in 100,000 annually.^16^ Both GPA and COVID‐19 exhibit a significant pulmonary involvement. GPA presents with a broad spectrum of clinical symptoms, leading to occasional oversight or delay in diagnosis. The onset of GPA following COVID‐19 infection is exceedingly rare.^17^

We report a case of GPA in an adolescent boy that developed subsequent to COVID‐19 infection. This constitutes the first documented case of GPA with DAH in an adolescent boy from Hunan Province, China, arising shortly after COVID‐19 infection, and presenting with pneumonia and DAH. The condition responded promptly to steroids, rituximab, and plasmapheresis. We summarized the clinical characteristics of new‐onset GPA post‐COVID‐19 infection by conducting a comprehensive literature review across databases such as PubMed, Wanfang Data, and CNKI, along with standard searches in Google Scholar, Cochrane, Scopus, and LitCovid.

Results indicated that cough and dyspnea were the most commonly observed clinical manifestations, with gastrointestinal bleeding and DAH being rare. In these patients, both c‐ANCA and PR‐3 antibodies were universally positive. CT findings in GPA were varied and included consolidation, multiple nodules, ground‐glass opacities, and cavitary nodules, with multiple nodules being the most prevalent. These features were not significantly different from usual GPA courses. Post‐COVID‐19 GPA cases had a longer duration of illness, which was considered to be related to the masking of the final diagnosis by COVID‐19 infection. Renal involvement was observed in over half of the patients. The majority received methylprednisolone and rituximab, with early diagnosis and treatment associated with a favorable prognosis. The final death of this patient was considered to be related to the lack of early diagnosis and treatment. The clinical manifestations of GPA in the early stage of its development are similar to those of COVID‐19 infections, which can be manifested only as fever and cough, etc., which can easily be misdiagnosed by doctors who do not have relevant experience in treating the disease, thus delaying the treatment of the patient and affecting the prognosis; the other factor influencing the poor prognosis of this patient was the combination of DAH and pulmonary embolism, which made the disease progress rapidly and increased the difficulty of treatment, resulting in the patient's death. Clinicians often encounter challenges in distinguishing GPA from COVID‐19 pneumonia due to their overlapping clinical and radiological features. Previous case reports have indicated that patients with unrecognized GPA, when treated for COVID‐19 pneumonia with steroids, show improvement, thereby masking the actual diagnosis.^17^ This suggests that, particularly during epidemics, new‐onset GPA may easily be misdiagnosed as pneumonia. The COVID‐19 pandemic has further complicated the diagnosis of newly identified GPA. Consequently, the use of clinical data and serologic tests is recommended to distinguish between COVID‐19 infection and underlying GPA.

GPA is a form of AAV characterized by a diverse array of clinical, imaging, and laboratory findings.^18^ While the precise mechanisms underlying GPA development remain elusive, a variety of factors, including genetics, medications, infections, and environmental exposures, are thought to contribute to its pathogenesis through the induction of autoimmunity.^1^, ^2^ Following the onset of the COVID‐19 pandemic, multiple reports have documented instances of AAV post‐COVID‐19,^19^ implicating COVID‐19 as a potential trigger for the onset of AAV.

Currently, two hypotheses exist regarding the mechanism by which COVID‐19 may induce GPA. The first hypothesis posits that autopsies of patients affected by SARS‐CoV‐2 have shown vasculitis in small, medium, and large vessels across multiple organ systems.^20^ It is speculated that the virus may directly invade endothelial cells, leading to vasculitis.

The second hypothesis considers the possibility that SARS‐CoV‐2 infection may act as a “trigger” for vasculitis, an aspect that has garnered significant interest within the debate surrounding COVID‐19 and GPA. Initially, the temporal association between COVID‐19 infection and the onset of GPA symptoms suggests a potential causal link. As detailed by Garlapati et al.,^21^ numerous viruses have been demonstrated to potentially predispose to GPA by elevating serum levels of inflammatory mediators. It is also known that SARS‐CoV‐2 elevates levels of these mediators, some of which are implicated in the pathogenesis of GPA. Consequently, an excess of inflammatory mediators may provide the necessary conditions for neutrophil priming and ANCA‐induced degranulation, potentially culminating in the development of GPA.

Postvaccination with SARS‐CoV‐2 messenger RNA vaccines, AAV has been reported.^22^, ^23^ The occurrence of AAV following SARS‐CoV‐2 infection and vaccination lends further support to the theory that the immune response to SARS‐CoV‐2 may act as a catalyst for the onset of GPA.

The immunological origins of GPA are complex, with approximately 80% of GPA patients testing positive for PR3‐ANCA.^24^ While a positive ANCA test is not essential for the clinical diagnosis of GPA, it is pathogenic and plays a pivotal role in the disease's etiology. According to various studies, ANCA titers have been observed to increase in the context of COVID‐19. Lee et al.^25^ assessed serum levels of myeloperoxidase (MPO)‐ANCA and PR3‐ANCA in 178 patients with SARS‐CoV‐2. They identified 22 cases of MPO‐ANCA (12.4%) and 14 cases of PR3‐ANCA (7.9%). GPA was subsequently diagnosed in 12 of these patients (6.7%). They concluded that SARS‐CoV‐2 infection may elevate ANCA positivity rates. Given that the prevalence of ANCA positivity in the general population stands at 0.9%, its prevalence among COVID‐19 patients is significantly higher.

In COVID‐19, SARS‐CoV‐2 binds to angiotensin‐converting enzyme 2, leading to the invasion of endothelial cells and subsequent microvasculitis. The persistent elevation of inflammatory cytokines and coagulation markers during and postinfection is associated with a sustained inflammatory response and a hypercoagulable state, contributing to the development of vascular diseases and other complications.^8^ In AAV, cytokine and macrophage‐induced inflammation leads to microvasculitis and endothelial damage. Additionally, some studies speculate a cross‐reaction between SARS‐CoV‐2 antigens and autoantibodies in autoimmune diseases.^26^ These findings suggest a potential causal relationship between SARS‐CoV‐2 infection and the onset of vasculitis.

The impact of SARS‐CoV‐2‐induced changes in nasopharyngeal and pulmonary microbiota on the manifestation of GPA in post‐COVID‐19 patients warrants consideration. Substantial evidence indicates that SARS‐CoV‐2 can alter the microbiome, and microbial dysbiosis is significantly correlated with elevated levels of tumor necrosis factor‐α, a key mediator in the formation of granulomatous lesions.^27^

## 7 CONCLUSION

We report a case of GPA accompanied by DAH, which manifested shortly following a COVID‐19 infection. COVID‐19 and newly onset GPA induced by COVID‐19 exhibit numerous overlapping clinical and radiological characteristics, posing significant challenges for clinicians in differentiating the two conditions. Additionally, our study offers insights into the diagnostic challenges associated with COVID‐19‐induced GPA. The limited case number and the retrospective nature of this review constitute its primary limitations. The retrospective analysis of this case could yield valuable insights into the assessment of clinical symptoms, diagnostic approaches, and management strategies for this perplexing condition. Furthermore, the pathophysiology and mechanisms underlying COVID‐19‐induced GPA remain incompletely understood and require further investigation. The potential for a shared immune mechanism underlying vasculitis development during SARS‐CoV‐2 infection and vaccination merits additional exploration. Such research could elucidate critical aspects of GPA pathogenesis and immune responses to COVID‐19, pivotal for both diagnosing and treating GPA.

## AUTHOR CONTRIBUTIONS

*Investigation*: Rong Jiang, Haibo Zhou, Long Wen, Xianglong Kong, Zhiguo Zhou. *Validation*: all declared authors. *Writing—original draft*: Rong Jiang. *Writing—review & editing*: Rong Jiang, Zhiguo Zhou. All authors read and approved the final manuscript.

## CONFLICT OF INTEREST STATEMENT

The authors declare no conflicts of interest.

## ETHICS STATEMENT

This study obtained ethical permission from the Science and Ethics Committee of Changsha First Hospital.

## Data Availability

The data presented in the study are included in the article/Supporting Information Material, further inquiries can be directed to the corresponding author.
